# Surgical repair of a large ventral hernia under spinal anaesthesia: A case report

**DOI:** 10.1016/j.amsu.2019.03.005

**Published:** 2019-03-16

**Authors:** Jurij Janež, Jasna Preskar, Matic Avguštin, Zdravko Štor

**Affiliations:** a University Medical Centre Ljubljana, Department of Abdominal Surgery, Ljubljana, Slovenia; b University of Ljubljana, Medical Faculty, Ljubljana, Slovenia

**Keywords:** Large ventral hernia, Surgical repair, Spinal anaesthesia, Rives-Stoppa technique

## Abstract

**Backround:**

Secondary ventral hernias are incisional hernias developed in former postoperative scars. Up to 30% of all patients undergoing laparotomy develop an incisional hernia. Open ventral hernia repair is often performed under general anaesthesia but can also be performed under regional anaesthesia.

**Case report:**

We report the case of an elderly man, who underwent open surgery of a large incisional hernia in spinal block. Regional anaesthesia was chosen due to the patient's additional diseases and disorders.

**Conclusion:**

Open surgery of large ventral hernia in spinal anaesthesia can be performed because the spinal anaesthesia provides adequate conditions for ventral hernia repair. The patient has to be in good physical condition in order for the surgery to be successful. During the surgery the patient has to be watched over vigilantly by the anaesthesiologist.

## Highlights

• Original case report.
• A rare case, not published previously in the literature.
• In literature there are rare descriptions of ventral hernia operations under spinal anaesthesia.
• Quality intraoperative images.

## 1 Introduction

A ventral hernia is defined as a fascial defect located on the abdominal wall. Primary ventral hernias are classified as umbilical, epigastric, Spigelian and lumbar hernias. There are also secondary ventral hernias which are incisional hernias developed in former postoperative scars [1]. Incisional hernia is the most frequent surgical complication after laparotomy. Up to 30% of all patients undergoing laparotomy develop an incisional hernia [2]. The elements that facilitate the development of incisional hernias are wound infections, corticosteroids, chemotherapy, radiation, insufficient nutrition and general over-stressing of the abdominal wall in the early stages of rehabilitation. The majority of incisional hernias occur in the first five years’ post-surgery. If a patient is able to undergo the surgery procedure including anaesthesia, the hernia is surgically repaired [3]. The repair of incisional hernia can be laparoscopic or open [2]. Defects may be closed with a simple figure-of-eight suture or with prosthetic mesh repair [4]. Open ventral hernia repair is often performed under general anaesthesia but can also be performed under regional anaesthesia [5]. Patients who are unable to undergo surgery can find relief by using hernia belts [3]. We present a case of an 80-year-old male patient, in whom a surgical repair of a large ventral hernia under spinal anaesthesia was performed.

This work has been reported in line with the SCARE 2018 criteria [6]. This work is registered with the Research Registry and the unique identifying number (UIN) is: researchregistry4745.

## 2 Case presentation

**Fig. 1:**
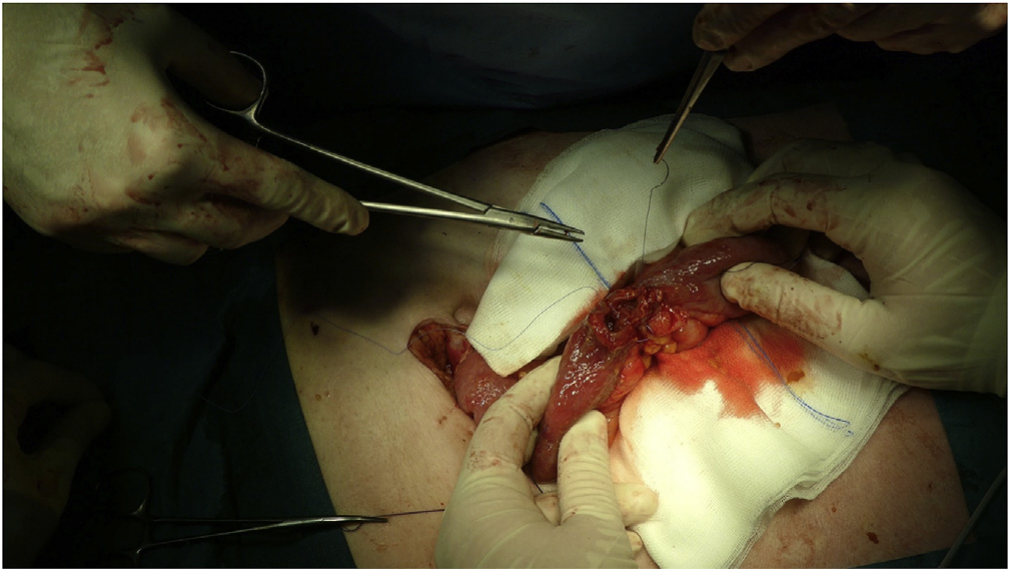
Segmental resection of a damaged small bowel with end-to-end anastomosis.

**Fig. 2:**
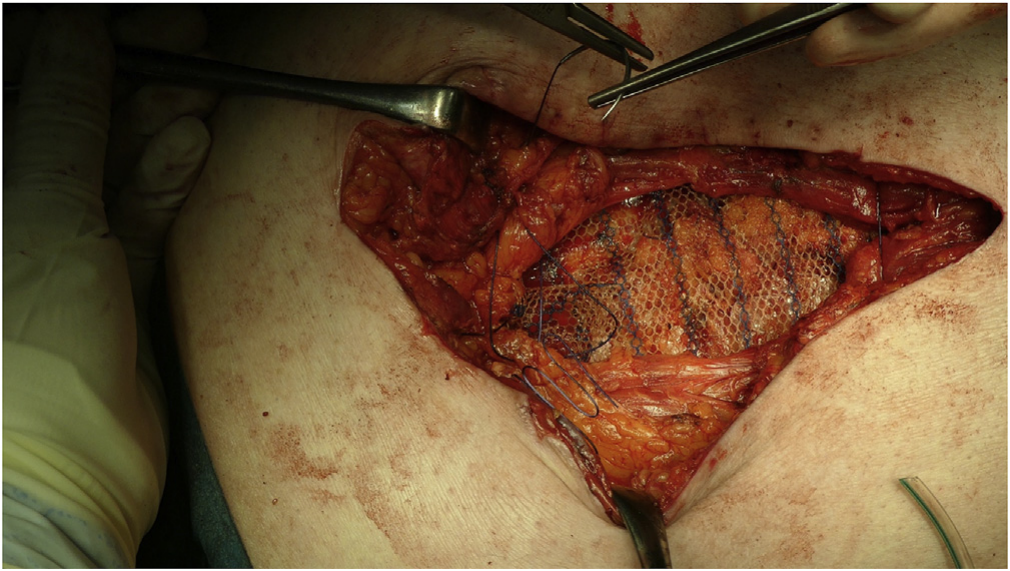
Rives-Stoppa hernioplasty with a prosthetic retromuscular mesh placement.

An 80-year-old male patient was referred to the abdominal surgery department due to incarcerated ventral hernia and ileus. In the past he was operated due to perforated gastric ulcer. He also had arterial hypertension, chronic pulmonary obstructive disease and pulmonary hypertension, a history of smoking, he suffered an ishemic stroke in the past. He was urgently operated on the same day. Segmental resection of small bowel with end-to-end anastomosis was performed and the hernia defect was closed with direct sutures, without prosthetic mesh because the bowel was resected. There were no surgical or other complications after surgery and he was discharged from hospital after 8 days. 5 days later he was admitted to the hospital again due to early recurrence of ventral hernia. The content in hernia sac could however be reduced back to his abdomen. Laboratory findings showed leucocytosis and elevated C-reactive protein (CRP - 148 mg/l). Intestinal winding with a thickened wall up to 5 mm was found at the location of the ventral hernia by ultrasound examination. The patient underwent a second surgery 22 days after the first surgery due to obstructive ileus, which was seen on the abdominal computed tomography (CT) a day earlier. Due to additional diseases and disorders (ischemic stroke and insertion of stent in his left internal carotid artery in 2011, arterial hypertension, asthma, pulmonary fibrosis and hypertension, which were not properly treated, because the patient did not follow the prescribed treatment) the anaesthesiologist decided for the spinal anaesthesia, because the general anaesthesia would be to risky. The surgery was performed by an abdominal surgeon with 5 years experiences as a specialist and he performed more than 30 Rives-Stoppa ventral hernia repairs. The skin incision was made along the previous skin incision. In the subcutaneous tissue the small intestine was tightly adhered on to the skin. We managed to release it but unfortunately, a segment of the small intestine was damaged during adhesyolisis. Segmental resection of the damaged small bowel with end-to-end anastomosis was performed (Fig. 1). The small intestine was reduced back in to the abdominal cavity. Ventral hernia was repaired according to Rives-Stoppa technique with prosthetic mesh (Fig. 2). Other than postoperative tachycardia there were no reported issues. A couple of hours after the procedure apnoeic episodes appeared followed by unconsciousness. A computed tomographic angiography (CTA) of the brain vascular system was made and it showed a stenotic left vertebral artery (90% stenosis). Because of respiratory insufficiency and haemodynamic instability, the patient was transferred to the intensive care unit. Due to a worsening clinical condition, a CTA of the abdomen was preformed and an occlusion of superior mesenteric artery (SMA) was discovered. Interventional radiologist preformed an embolectomy and thrombus aspiration from the SMA with an insertion of a stent. The patient's condition continued to worsen so the abdominal surgeon decided for a “second look” abdominal exploration. At surgical revision we found a small intestinal and sigmoid colon gangrene. Because of the patients age, several other comorbidities and gangrene of the entire small bowel, the multidisciplinary team (abdominal surgeon, anaesthesiologist, intensivist) decided for conservative treatment. The patient died the day after surgery.

## 3 Discussion

Ventral hernias are common post-surgical complications of laparotomy, up to 30% of all patients develop an incisional hernia [2]. One of the elements that facilitate the development of incisional hernias is general over-stressing of the abdominal wall in the early stages of rehabilitation. The patient in question was coughing frequently due to an untreated pulmonary disease and a history of smoking. He was also elderly and obese and had pulmonary fibrosis. The before-mentioned facts contributed to the development of the incisional hernia which was likely to occur even under better circumstances [7]. During the first surgery patient was not treated by mesh prosthesis because of bowel resection, which is a surgical procedure with the lowest recurrence rate [4,7]. A non-incarcerated hernia usually does not require surgery. An evaluation of the patient's issues with hernia in regard with possible anaesthesia and surgery complications is necessary to determine whether a patient should undergo surgery. Due to the patient's additional diseases and disorders, especially the respiratory ones, general anaesthesia would be highly risky [8]. Questions were also raised regarding the potential benefits of surgery due to his conditions and a likelihood of recurrence [7]. A decision was made to postpone the surgery. The patient stayed in the hospital for observation. Because of the patient's discomfort with breathing and abdominal pain, a CT of the abdomen and thorax was performed. Obstructive ileus of small bowel was found. Because of large ventral hernia and lack of abdominal musculature his respiratory function was additionally compromised. A multidisciplinary team (abdominal surgeon, anaesthesiologist, internal medicine specialist) decided for a surgery and attempt of hernia repair. The anaesthesiologist decided for spinal block because general anaesthesia (GA) was considered too risky. Spinal anaesthesia (SA) provides safe and reliable surgical anaesthesia in adult patients undergoing elective open ventral hernioplasty [5,9]. The crucial advantage for the patient in question is avoidance of airway instrumentation. The lack of instrumentation prevents an attack of bronchospasm [8,10]. Use of agents that could depress the respiratory system (i.e. opiates) cannot be precluded during SA but is lower than in GA [11] which is another advantage for patients with respiratory conditions. Other advantages of spinal anaesthesia include better postoperative pain control and less post-operative nausea and vomiting (PONV), reduction of the need for blood transfusion, lower incidence of thromboembolic disease, pulmonary embolism, postoperative hypoxic episode and lower total drug and supply cost [5]. Some reports suggest, that this procedure may be more beneficial in the patients, which are at high risk for GA due to presence of other diseases [10]. There are several disadvantages of the spinal anaesthesia due to sympathetic denervation of high regional block, which might lead to bradycardia, hypotension and decreased cardiac output. If the spinal block is to high, respiratory function can be compromised due to paralysis of respiratory musculature. Vigilant monitoring during and after surgery is essential for prevention and treatment of complications of SA [5,10]. One of the feared complications in abdominal surgery under regional anaesthesia is inadequate muscle relaxation. The reason turned out to be unjustified as the anaesthesiologist provided sufficient muscle relaxation just as suggested in some reports [12]. Furthermore, a body mass index (BMI) of more than 25 kg/m2 represents an independent risk factor for spinal block failure. The rate of spinal block failure seems to increase incrementally with BMI [13]. This is an important observation for the patient in question and other patients with hernias as they are often obese. The surgeon and anaesthesiologist must be well prepared for the surgery as well as the patient who must be mentally and physically fit. Anxiety or pain during regional anaesthesia could precipitate an attack of bronchospasm [8]. Additional diseases or disorders should be under control, additional testing depending on the patient's age and diseases should be taken into consideration [14]. We advise a healthy life style. Patients who undertake a period of short‐term preoperative smoking cessation may reduce their risk of postoperative complications in comparison with patients who continue to smoke [15]. Weight reduction for obese patients is also advised. After ventral hernia repair, complications are most likely to occur in patients with BMI ≥40 kg/m2 [16]. The patient in question was unfortunately not in optimal shape for the surgical procedure.

Another issue regarding the surgical technique in our patient is the use of prosthetic mesh. Use of a mesh with intraoperative bowel injury is controversial, however due to a large abdominal wall defect, there was no other option to repair the hernia. At first operation the mesh was not used because of intraoperative bowel injury and segmental resection, but the patient had an early recurrence. This was another reason for our decision to perform hernia repair with prosthetic mesh.

## 4 Conclusion

Open surgery of large ventral hernia in spinal anaesthesia can be performed because the spinal block provides adequate anaesthesia and is an alternative option for patients who are at high risk for GA. The crucial advantage for the patient is avoidance of airway instrumentation. During the surgery the patient has to be watched over vigilantly by the anaesthesiologist because sympathetic denervation presents a risk for haemodynamic complications. The patient has to be in good physical condition in order for the surgery to be successful. All of the above can be achieved with fewer risk by elective surgeries, because they are planned months in advance.

Our case described a surgical repair of a large ventral hernia under spinal anaesthesia. The surgery was performed without complications, but the patient unfortunately died because of thrombembolic complication, which was most likely connected with patients several comorbidities. Our case showed, that surgical repair of ventral hernia is feasible under spinal anaesthesia but is probably more appropriate for small or medium sized hernias and for patients, who are otherwise healthy, because of the reduced risk of postoperative complications.

## Sources of funding

This work received no funding.

## Provenance and peer review

Not commissioned, externally peer reviewed.

## Ethical approval

This work is a case report and in our country does not need an ethical approval.

## Author contribution

JJ and MA performed the second surgery, MA performed the first surgery. JP and JJ wrote the first draft of the manuscript and ZŠ contributed to data curation, validation, visualization and writing and editing of the manuscript.

## Conflicts of interest

The authors (JJ, JP, MA, ZŠ) declare no conflicts of interests or disclosures.

## Trial registry number

Researchregistry4745.

## Guarantor

Jurij Janež.

## Appendix A Supplementary data

SCARE 2018 SCARE 2018

## References

[1] Eriksen J.R. (2001 Dec). Pain and convalescence following laparoscopic ventral hernia repair. Dan. Med. Bull..

[2] Eker H.H., Hansson B.M.E., Buunen M. (2013 Mar). Laparoscopic vs open incisional hernia repair: a randomized clinical trial. JAMA Surg [Internet].

[3] Smrkolj V. (2014). Kirurgija.

[4] Burger J.W., Luijendijk R.W., Hop W.C., Halm J.A., Verdaasdonk E.G., Jeekel J. (2004 Oct). Long-term follow-up of a randomized controlled trial of suture versus mesh repair of incisional hernia. Ann Surg [Internet].

[5] Krobot R., Premužić J. (2013). Comparison of general and spinal anaesthesia in patients undergoing open ventral hernia repair. Periodicum biologorum [Internet].

[6] Agha R.A., Borrelli M.R., Farwana R., Koshy K., Fowler A.J., Orgill D.P., For the SCARE Group (2018). The SCARE 2018 statement: updating consensus surgical CAse REport (SCARE) guidelines. Int. J. Surg..

[7] Toyoshima H. (1986 Jul). Surgery of incisional hernia and its pro gnosis–statistical analysis in 657 patients. Nippon. Geka Gakkai Zasshi.

[8] Woods B.D., Sladen R.N. (2018 Dec). Perioperative considerations for the patient with asthma and bronchospasm. BJA [Internet].

[9] Sultan S., Rana S.S., Hafeez A., Tariq U. (2018). Comparison of general and spinal anesthesia in patients undergoing open abdominal hernia repair in terms of post-operative pain. APMC.

[10] Dhumane P., Naqeeb Mujtaba T.I.S. (2016 Apr). Efficacy of spinal anaesthesia for laparoscopic ventral hernia repair. IJBR [Internet].

[11] Symeonidis D., Baloyiannis I., Georgopoulou S., Koukoulis G., Athanasiou E., Tzovaras G. (2013 Jul 13). Laparoscopic ventral hernia repair in obese patients under spinal anesthesia. Int J Surg [Internet].

[12] Sinha R., Gurwara A.K., Gupta S.C. (2008 Apr-Jun). Laparoscopic surgery using spinal anesthesia. J. Soc. Laparoendosc. Surg..

[13] Cotter J.T., Nielsen K.C., Guller U. (2004 Oct). Increased body mass index and ASA physical status IV are risk factors for block failure in ambulatory surgery - an analysis of 9,342 blocks. Can J Anesth [Internet].

[14] Barnard N.A., Williams R.W., Spencer E.M. (1994 Sep). Preoperative patient assessment: a review of the literature and recommendations. Ann. R. Coll. Surg. Engl..

[15] Theadom A., Cropley M. (2006 Oct). Effects of preoperative smoking cessation on the incidence and risk of intraoperative and postoperative complications in adult smokers: a systematic review. Tob Control [Internet].

[16] Pernar L.I., Pernar C.H., Dieffenbach B.V., Brooks D.C., Smink D.S., Tavakkoli A. (2017 Mar). What is the BMI threshold for open ventral hernia repair?. Surg Endosc [Internet].

