# Supplementary material for: Surgical repair of a large ventral hernia under spinal anaesthesia: A case report
Source: Ann Med Surg (Lond). 2019 Mar 16;40:31–3. doi: 10.1016/j.amsu.2019.03.005 (PMC6430731; doi:10.1016/j.amsu.2019.03.005)
Supplement: SCARE 2018 [file mmc1.pdf]

SCARE 2018 Checklist

Topic	Item	Checklist item description	Page Number
**Title**	**1**	The words "case report" should appear in the title. The title should also describe the area of focus (e.g. presentation,	1
**Key Words**	**2**	3 to 6 key words that identify areas covered in this case report (include "case report" as one of the keywords)	1
**Abstract**	**3a**	Introduction — Describe what is unique or educational about the case (i.e. what does this work add to the surgical literature, and why is this important?).	1
	**3b**	Presenting complaint and investigations – describe the patient's main concerns and important clinical findings.	1
	**3c**	The main diagnoses, therapeutics interventions, and outcomes.	1
	**3d**	Conclusion — Describe the main lessons to "take-away" from this case study	1
**Introduction**	**4**	Background – summarise what is unique or educational about the case. Give reference to the relevant surgical literature and current standard of care. The background should be referenced, and 1-2 paragraphs in length.	2
**Patient Information**	**5a**	Demographic details – include de-identified demographic details on patient age, sex, ethnicity, occupation. Where possible, include other useful pertinent information e.g. body mass index and hand dominance.	2
	**5b**	Presentation - describe the patient's presenting complaint (symptoms). Describe the patient's mode of presentation (brought in by ambulance or walked into Emergency room or referred by family physician).	2
	**5c**	Past medical and surgical history, and relevant outcomes from interventions	2

	5d	Other histories – Describe the patient's pharmacological history including allergies, psychosocial history (Drug, smoking, and if relevant, accommodation, walking aids), family history including relevant genetic information.	2
**Clinical Findings**	6	Describe the relevant physical examination and other significant clinical findings. Include clinical photographs where relevant and where consent has been given.	2
**Timeline**	7	Inclusion of data which allows readers to establish the sequence and order of events in the patient's history and presentation (using a table or figure if this helps). Delay from presentation to intervention should be reported.	2
**Diagnostic Assessment**	8a	Diagnostic methods – describe all investigations taken to arrive at methods: physical exam, laboratory testing, radiological imaging, histopathology.	2, 3
	8b	Diagnostic challenges – describe what was challenging about the diagnoses, where applicable, for example access, financial, cultural.	2, 3
	8c	Diagnostic reasoning – Describe the differential diagnoses and why they were considered.	2, 3
	8d	Prognostic characteristics when applicable (e.g. tumour staging or for certain genetic conditions). Include relevant radiological or histopathological images in this section.	
**Therapeutic Intervention**	9a	Pre-intervention considerations – if there were patient-specific optimisation measures taken prior to surgery or other intervention these should be included e.g. treating hypothermia/hypovolaemia/hypotension in a bums patient, Intensive care unit treatment for sepsis, dealing with anticoagulation/other medications, etc.	2
	9b	Interventions – describe the type(s) of intervention(s) deployed (pharmacologic, surgical, physiotherapy, psychological, preventive). Describe the reasoning behind this treatment	2, 3

		offered. Describe any concurrent treatments (antibiotics, analgesia, anti-emetics, nil by mouth, Venous thrombo-embolism prophylaxis, etc). Medical devices should have manufacturer and model specifically mentioned.	
	**9c**	Intervention details – describe what was done and how. For surgery include details on; anaesthesia, patient position, use of tourniquet and other relevant equipment, prep used, sutures, devices, surgical stage (1 or 2 stage, etc). For pharmacological therapies include information on the formulation, dosage, strength, route, duration, etc. Include intra-operative photographs and/or video or relevant histopathology in this section. Degree of novelty for a surgical technique/device should be mentioned e.g. "first in human".	2,3
	**9d**	Who performed the procedure - operator experience (position on the learning curve for the technique if established, specialisation and prior relevant training). For example, "junior resident with 3 years of specialised training"	2
	**9e**	Changes – if there were any changes in the interventions, describe these details with the rationale.	3
**Follow-up and Outcomes**	**10a**	Follow-up – describe 1) When the patients was followed up. 2) Where. 3) How (imaging, tests, scans, clinical examination, phone call), and 4) whether there were any specific post-operative instructions. Future surveillance requirements - e.g. imaging surveillance of endovascular aneurysm repair or clinical exam/ultrasound of regional lymph nodes for skin cancer.	3
	**10b**	Outcomes - Clinician assessed and (when appropriate) patient-reported outcomes (e.g. questionnaire details). Relevant photographs/radiological images should be provided e.g. 12 month follow-up.	3
	**10c**	Intervention adherence/compliance - where relevant how well patient adhered to and tolerated their treatment. For example,	2,3

		post-operative advice (heavy lifting for abdominal surgery) or tolerance of chemotherapy and pharmacological agents	
	10d	Complications and adverse events – all complications and adverse or unanticipated events should be described in detail and ideally categorised in accordance with the Clavien-Dindo Classification. How they were prevented, diagnosed and managed. Blood loss, operative time, wound complications, re-exploration/revision surgery, 30-day post-op and long-term morbidity/mortality may need to be specified. If there were no complications or adverse outcomes this should also be included.	3
Discussion	11a	Strengths – describes the strengths of this case	3,4
	11b	Weaknesses and limitations in your approach to this case. For new techniques or implants - contraindications and alternatives, potential risks and possible complications if applied to a larger population. If relevant, has the case been reported to the relevant national agency or pharmaceutical company (e.g. an adverse reaction to a device)	3,4
	11c	Discussion of the relevant literature, implications for clinical practice guidelines and any relevant hypothesis generation.	3,4
	11d	The rationale for your conclusions.	3,4
	11e	The primary “take-away” lessons from this case report.	4
Patient Perspective	12	When appropriate the patient should share their perspective on the treatments they received.	4
Informed Consent	13	Did the patient give informed consent for publication? Please provide if requested by the journal/editor. If not given by the patient, explain why e.g. death of patient and consent provided by next of kin or if patient/family untraceable then document efforts to trace them and who within the hospital is acting as a guarantor of the case report.	5

**Additional Informatio n**	**14**	Conflicts of Interest, sources of funding, institutional review board or ethical committee approval where required.	4
